# Current Concepts in Robotics for the Treatment of Joint Disease

**DOI:** 10.1155/2013/948360

**Published:** 2013-12-23

**Authors:** Michael A. Conditt, William L. Bargar, Justin P. Cobb, Lawrence D. Dorr, Jess H. Lonner

**Affiliations:** ^1^MAKO Surgical Corporation, USA; ^2^The Sutter Joint Replacement Center, 1020 29th Street No. 450, Sacramento, CA 95816, USA; ^3^Department of Orthopaedics, Academic Health Science Centre, Charing Cross Hospital, Imperial College, UK; ^4^The Arthritis Institute, USA; ^5^Rothman Institute, Thomas Jefferson University, Philadelphia, USA

## 1. Introduction 

Adoption of a new technology in surgery today is subject to assessment by many stakeholders. These include surgeons, patients, hospitals, regulators, and payers. The fundamental tool for assessment is the determination of “value.” But value has different meanings for each of the stakeholders. The usual definition of value is “outcome divided by cost.” Although cost is usually measured in dollars, the measures for “outcome” are not clearly defined nor agreed upon. What follows is an attempt to define the value of robotic surgery in joint replacement surgery for each of the stakeholders.

First, however, we need to understand that the primary value of robotics in joint replacement is the reduction of human error by improving accuracy and precision. This is the same value that has resulted in adoption of robotics in most manufacturing processes. A major part of quality control in manufacturing is optimizing accuracy and precision by reducing human error. Surgery, however, is a blend of intelligence, art, and skill. There are many human skills that are poorly performed by robots and vice versa. The appropriate use of robotics in joint replacement surgery is intended to improve the accuracy and precision of implant selection and placement as well as execution by bone preparation. The goal is not to replace the surgeon but to enhance the surgeon's performance. Robotics offers a tool that enables the surgeon to reproduce his/her best performance on a consistent basis.

## 2. The Surgeon

Surgeons assess the value of new technology in terms of the outcome of their patients as well as the effect on their practice. The academic assessment of patient outcome has seen a shift over the last 10+ years from surgeon-assessed measures (like the Harris Hip Score or original Knee Society Score) to patient-assessed measures (like the WOMAC or SF-36). Recently, it has been recognized that more sensitive measures like visual analog pain scales and patient satisfaction surveys are needed to assess differences in higher levels of function. Although surgeons follow the publications of these academic outcome studies, the practical assessment of patient outcome by the surgeon is a much more subjective process that is both surgeon specific and practice specific.

Surgeons are concerned with performance. In this way they are like professional athletes. Golfers prepare by using “positive swing thoughts,” and quarterbacks focus on execution of a pass play not the last time they threw an interception. Similarly, surgeons focus on how to achieve the best performance for each surgery they attempt. As they mentally prepare for their performance, they necessarily focus on their past successful surgeries. And, just like the athletes, they tend not to focus on their prior mistakes or failures. This is necessary and effective prior to and during the performance of a surgery. After the surgery, however, the best surgeons and athletes analyze their performance critically. They acknowledge their mistakes and determine ways to prevent them in the future. But most surgeons, like most athletes, tend to gloss over their mistakes and remember their successes. This makes it difficult for them to determine the value of a technology like robotics that is designed to reduce human error.

The economic impact of adopting robotics on the surgeon's practice has potentially both a positive and a negative effect. By using a technology that improves the accuracy and precision of their surgeries, they may attract more patients. Some surgeries, however, at present require longer operating times (10–20 min). This can have a negative economic effect if the surgeon is doing many joint replacements in a single day. But, for most surgeons doing only a few joint replacements in one day, the extra time would not allow the addition of another case. There is also additional time required for preop planning for robotic surgery (10–15 min). But, again, for the usual surgeon doing only a few cases each week, this additional time may not be significant.

## 3. The Patient

Cost is usually not an issue for the patient when determining value, since most are covered by insurance and usually no additional charges are passed on to the patient. Patients, however, obviously want the best outcome. Outcome can mean different things to each patient. Indeed, a large part of obtaining an informed consent from a patient is explaining to them what to expect in terms of pain, function, and limitations as well as reviewing the usual risks. What about the risk of using robotics?

There is an inherent fear of robots on the part of patients. In part, this is due to Hollywood movies and their fascination with robots that go crazy and cause havoc. But patients are also concerned that robots, like computers, may have “bugs” in the programming or “crash” like their home computers. Will the robot go crazy? This is where it is helpful to explain to the patient how the development of robots in industry over the last 30+ years has virtually eliminated robot error by the use of redundancy and internal safety checks. The engineers designing robots take Isaac Asimov's First Law of Robotics seriously: a robot is not allowed to harm a human. Only human error can result in robot error. In this case, the human is the surgeon. In robotic surgeries, the surgeon has some very important responsibilities: select the best size and type of implant for the patient, position it appropriately, and provide the robot with a workspace such that no soft tissues are damaged.

It is quite a big step for a patient to surrender his or her body to a surgeon for an invasive procedure. It is also a very subjective and emotional decision for the patient. Once they have decided to have the surgery, they want to have faith in their surgeon. They want to believe that their surgeon is the best. Again, this presents a problem when telling patients about using a technology that reduces human error. They really do not like to think about their surgeon making errors. Here, another reference to sports may help. If you ask the patient who the best golfer in the world is, and then ask if he always hits the ball in the middle of the fairway, they will realize that even the best human skills are subject to error. In this way, they will be much more receptive to the use of robotic technology.

## 4. The Hospital

In my experience, hospitals are only concerned about patient outcome in so far as it might relate to complications that arise within the first 90 days. For hip replacement, robotics can help in reducing early dislocations as well as intraoperative fractures, but otherwise the use of this technology offers little to reduce short-term complications.

Hospitals are most interested in cost and return on investment “ROI.” Robotic systems can cost the hospital more than $1 million. There are some small potential savings for the hospital in terms of reduction of inventory and less use of conventional instruments, but these “soft costs” are difficult to measure. The true beneficiaries of reductions in inventory and instruments are the implant manufacturers. In the future, the hospital may be able to negotiate a lower price for implants used in robotic surgeries.

The cost of the technology to the hospital is mainly offset, however, by attracting new patients and “filling beds,” preferably with non-Medicare and noncapitated patients. There is also the so-called “halo effect” where new technology can attract patients that have other health problems which can result in increased admissions and testing for other conditions. If a preop CT scan is done (which is required by some robotic systems for preoperative planning and mapping), this may either be an additional source of revenue or expense for the hospital depending on contracts with payors.

## 5. Regulators

The US FDA defines value in terms of safety and efficacy. These requirements were stipulated in the 1974 Medical Device Act which basically said that medical devices should be treated in the same way as pharmaceuticals. They established two different pathways to FDA clearance: the so-called 510K pathway and the “Premarket Approval” pathway. The 510K process is for devices that can be shown to be “substantially equivalent” to a device already approved by the FDA. The Premarket Approval process is for a new device with no substantial equivalence to a device already on the market. Most new orthopaedic devices are cleared by the 510K process, since the Premarket Approval process is very costly and usually takes many years. For robotic device manufacturers, the issue of showing substantial equivalence to an existing device has been problematic. In the past, the 510K process did not require new clinical data, whereas the Premarket Approval process requires randomized controlled clinical trials. Recently, however, there has been a trend to require clinical data for many 510K applications. This may be in response to significant problems with some 510K devices after they have been cleared and in general use (e.g., metal-on-metal hips).

Beginning in the late 1990s, a third requirement (in addition to showing safety and efficacy) has been added for clearance called “clinical utility.” This is not defined by the FDA. In fact, they usually ask the applicant to define the clinical utility of the device. Usually this has something to do with cost-effectiveness. The criteria for meeting the clinical utility standard are not clearly understood.

When it comes to evaluating the safety, efficacy, and clinical utility of robotics, there has been some confusion. Is a robot just a new more sophisticated tool to be used in surgery, like a smart reamer or saw? Are clinical data necessary? If so, how long should the patients be followed after surgery? If a complication arises, is it the tool that causes it, or is it the inappropriate use of that tool by the surgeon? Is there any real difference in control between semiactive (haptic) robots where the surgeon guides the tool but the limits of movement are restricted by the robot and active robots where the cut paths of the tool are guided by the robot with the same intended limits? These are all the questions under consideration by the FDA.

## 6. Payers

Usually insurance companies do little new technology assessment. If Medicare decides to cover something, they will usually follow suit. So far, there is no CPT code for the use of robotic surgery in joint replacement. Computer navigation does have a code, but reimbursement by Medicare has been spotty.

Payers should be most interested in reducing complications and improving longevity of the joint replacement. Readmissions and revisions of failed implants are very costly. Robotic joint replacement surgery offers the distinct possibility to reduce human error in surgery. Data supporting the reduction of complications or increased longevity of implants put in with robotics have been difficult to obtain. Without some data, some payers are unlikely to pay more for the use of this technology.

## 7. Our Opinion

The intrinsic value of using robotics to improve accuracy and precision in joint surgery will ultimately be recognized as adding significant value. We expect to see a surge of interest from surgeons in the future as new generations of robots with robust applications will address many, if not all, of the outstanding issues including ROI, reliability, better outcome, and operative times. With increasing emphasis on outpatient partial knee replacements, the role of robotic surgery in ambulatory surgery centers is a growing consideration and becoming a greater reality, particularly as pricing of systems improves. Current clinical applications of robotics in joint replacement will improve and different applications to other aspects of joint reconstructive surgery will be added.

*Michael A. Conditt* *William L. Bargar* *Justin P. Cobb* *Lawrence D. Dorr* *Jess H. Lonner*

